# Does maternal obesity during pregnancy impact offspring’s liver and skeletal muscle metabolism?

**Published:** 2022-12

**November 23, 2022** - A recent study in The FASEB Journal has identified metabolic perturbations in the liver and skeletal muscle of young nonhuman primates on normal diets whose mothers were obese during pregnancy.

For the study, tissue biopsies were obtained from 19 post-pubertal offspring of mothers who were fed a Western diet and were obese during pregnancy, and from 13 control animals born to non-obese mothers fed a standard diet. All offspring ate a healthy chow diet after weaning. Investigators identified 58 metabolites significantly altered in liver and 46 in skeletal muscle of the offspring of mothers with obesity during pregnancy, with 8 metabolites shared between both tissues. Several metabolic pathways were identified from these dysregulated metabolites. These differences in metabolites were not seen in blood samples taken from the animals.

“This study is exciting for two reasons: First it shows that exposure to an unhealthy environment in utero has long-term health consequences, and different organs and tissues are affected in different ways.” said corresponding author Michael Olivier, PhD, of Wake Forest University School of Medicine. “Second, our analysis suggests you cannot just analyze blood samples to understand what is happening in the liver or muscle.”

***Link to Study: https://onlinelibrary.wiley.com/doi/10.1096/fj.202201473R***

***Full citation:** “Maternal obesity alters offspring liver and skeletal muscle metabolism in early post-puberty despite maintaining a normal post-weaning dietary lifestyle.” Isaac Ampong, Kip D. Zimmerman, Danu S. Perumalla, Katharyn E. Wallis, Ge Li, Hillary F. Huber, Cun Li, Peter W. Nathanielsz, Laura A. Cox, Michael Olivier. FASEB J; Published Online: November 23, 2022 (DOI: 10.1096/fj.202201473R).*

*Copyright © 2019 The Cochrane Collaboration. Published by John Wiley & Sons, Ltd., reproduced with permission.*

